# Mouse models of SMA show divergent patterns of neuronal vulnerability and resilience

**DOI:** 10.1186/s13395-022-00305-9

**Published:** 2022-09-12

**Authors:** Victoria Woschitz, Irene Mei, Eva Hedlund, Lyndsay M. Murray

**Affiliations:** 1 Centre for Discovery Brain Sciences, College of Medicine and Veterinary Medicine, University of Edinburgh, Old Medical School, Teviot Place, Edinburgh, EH8 9XD UK; 2 Molecular, Genetic and Population Health Sciences, Usher Institute, University of Edinburgh, Edinburgh, EH16 4UX UK; 3 Euan McDonald Centre for Motor Neuron Disease Research, University of Edinburgh, Edinburgh, EH8 9XD UK; 4 Department of Biochemistry and Biophysics, Stockholm University, 106 91 Stockholm, Sweden; 5 Department of Cell and Molecular Biology, Karolinska Institutet, 171 77 Stockholm, Sweden

**Keywords:** Spinal muscular atrophy, Mouse model, Selective vulnerability, Neuromuscular junction, NMJ, Motor neuron, Degeneration, Transcriptional analysis, Gene ontology

## Abstract

**Background:**

Spinal muscular atrophy (SMA) is a form of motor neuron disease affecting primarily children characterised by the loss of lower motor neurons (MNs). Breakdown of the neuromuscular junctions (NMJs) is an early pathological event in SMA. However, not all motor neurons are equally vulnerable, with some populations being lost early in the disease while others remain intact at the disease end-stage. A thorough understanding of the basis of this selective vulnerability will give critical insight into the factors which prohibit pathology in certain motor neuron populations and consequently help identify novel neuroprotective strategies.

**Methods:**

To retrieve a comprehensive understanding of motor neuron susceptibility in SMA, we mapped NMJ pathology in 20 muscles from the *Smn*^*2B/-*^ SMA mouse model and cross-compared these data with published data from three other commonly used mouse models. To gain insight into the molecular mechanisms regulating selective resilience and vulnerability, we analysed published RNA sequencing data acquired from differentially vulnerable motor neurons from two different SMA mouse models.

**Results:**

In the *Smn*^*2B/-*^ mouse model of SMA, we identified substantial NMJ loss in the muscles from the core, neck, proximal hind limbs and proximal forelimbs, with a marked reduction in denervation in the distal limbs and head. Motor neuron cell body loss was greater at T5 and T11 compared with L5. We subsequently show that although widespread denervation is observed in each SMA mouse model (with the notable exception of the Taiwanese model), all models have a distinct pattern of selective vulnerability. A comparison of previously published data sets reveals novel transcripts upregulated with a disease in selectively resistant motor neurons, including genes involved in axonal transport, RNA processing and mitochondrial bioenergetics.

**Conclusions:**

Our work demonstrates that the *Smn*^*2B/-*^ mouse model shows a pattern of selective vulnerability which bears resemblance to the regional pathology observed in SMA patients. We found drastic differences in patterns of selective vulnerability across the four SMA mouse models, which is critical to consider during experimental design. We also identified transcript groups that potentially contribute to the protection of certain motor neurons in SMA mouse models.

**Supplementary Information:**

The online version contains supplementary material available at 10.1186/s13395-022-00305-9.

## Background

Spinal muscular atrophy (SMA) is a form of motor neuron disease affecting primarily children that is caused by mutations and deletions within the survival motor neuron 1 (*SMN1*) gene [1]. The reduced levels of survival motor neuron (SMN) protein cause progressive loss of lower motor neurons and atrophy of associated skeletal muscles [1–3]. A second partially functional copy of this gene (termed *SMN2*) can exist in varying copy numbers and is an important modifier of disease severity [4, 5]. Consequently, SMA can be classified into 4 subtypes based upon the age of onset and expected prognosis. The most severe and common form of SMA (type I) has an onset of 6 months or earlier and has—if untreated—a life expectancy of 2 years without significant respiratory support. Three treatments have now been approved for patients with SMA [6–11]. Despite the significant benefits that these can confer to patients, impactful motor deficits can remain, even when treatment is administered pre-symptomatically [12, 13]. In order to optimise treatment for all patients, it is imperative that we maintain momentum to understand the fundamental biology underlying SMA.

The central clinical manifestations of SMA are driven by the loss of lower motor neurons. Here, the synapses between motor neurons and muscles, neuromuscular junctions (NMJs), are early and important pathological targets [14], with NMJ loss coinciding with the onset of cell death pathways at the soma, but preceding a quantifiable loss of motor neurons [15, 16]. A wide range of morphological and functional defects of NMJs have previously been reported in mouse models of the disease [14, 17–20]. Although the sequential breakdown of motor neurons is relatively well defined, it has become apparent that not all motor neurons are uniformly vulnerable. Indeed, it has long been recognised that severe SMA patients have more profound weakness in the core and proximal limb muscles, with a comparative sparing of the muscles of the distal limbs, hands, feet and face [21]. Indeed, a more recent study examining muscle function using hammer-smith motor function scales found that muscle location was a predictor of relative weakness, wherein weakness was more profound in proximal limb muscles compared to distal limb muscles [22]. In type 3 SMA patients, patterns of vulnerability are once again highly consistent, with increased vulnerability of the triceps, iliopsoas, thigh adductors, and quadriceps [23].

All SMA mouse models examined thus far show dramatic intermuscular variability in the levels of NMJ loss observed [14, 20, 24–29]. This has been characterised in different mouse models of SMA, including the *Smn*^*-/-*^*;SMN2* (life expectancy 6 days) [14, 24, 25], *SMN*^*Δ7/Δ7*^*;SMN2* aka Taiwanese (life expectancy 8–10 days) [29] and *Smn*^*-/-*^*;SMN2;SMN*^*Δ7/Δ7*^ aka *SMNΔ7* (life expectancy 13 days) [14, 20, 28]. In addition, there is the *Smn*^*2B/-*^ mouse model, created through the mutation of a splice enhancing site in the murine *Smn* gene [30, 31]. Its life expectancy and severity varies depending on the genetic background [26, 27, 30–32], but in our hands, it has a phenotypic onset of 10 days and a life expectancy of 18 days [15]. Although NMJ pathology and selective vulnerability are evident in this model, only a small number of muscles have thus far been characterised [15, 27].

The reasons for differential vulnerability among motor neurons in SMA are currently unclear, but it is increasingly important that we understand the patterns and investigate their basis. Indeed, it is well established that early administration of Smn-dependant therapeutics confers the greatest benefit to patients, with reductions in efficacy observed as treatment is delayed [33]. We have recently shown the recovery of neuromuscular connectivity is proportionate to the time of treatment, but is also highly dependent upon the relative vulnerability of the muscle [34]. Electrophysiology in patients has shown that treatment can induce an increase in the number of active motor units which relate to gains in motor function [35]. Early findings would suggest that the gains in motor function are proportionate to the relative vulnerability of the muscle, and the time period since the motor function was lost. It is therefore critical to profile this selective vulnerability in experimental models and thus provide a platform for the development of future combinational therapeutics which will target the most vulnerable motor units.

The phenomenon of differential resilience also provides an opportunity to investigate mechanisms that drive motor neuron protection, which can reveal disease modifiers. This approach has been used to identify modifiers of motor neuron pathology in ALS. Here, motor neurons with differential susceptibility in ALS were isolated from post mortem tissues of neurologically normal mice, rats or humans and subjected to global transcriptome analysis. This led to the identification of a number of important modifiers, including IGF-II [36], GABA and glutamate receptors [37], MMP-9 [38] and synaptotagmin 13 [39]. In SMA, the same approach led to the important identification of DNA repair transcripts [27] and Pgk1 [40] as potential disease modifiers. In the former study, a four-way comparison of motor neurons which were vulnerable or resistant in wildtype and *Smn*^*2B/-*^ mice facilitated the identification of transcripts associated with a reduced *Smn* level and those specifically involved in the motor unit breakdown. A longitudinal study of transcriptional profiling on discrete neuronal populations at pre-, early- and late-symptomatic timepoints from wildtype and *SMN∆7* mice further identified potential modifiers of disease, including Gdf-11 [41]. Gene ontology and protein network analysis revealed unique disease-adaptation mechanisms present in resistant ocular motor neurons that could be responsible for their sparing. These motor neurons were found to upregulate transcripts associated with modulation of neurotransmitter release, neuronal survival and protection from oxidative stress. The study revealed cell-specific changes that present promising targets for the protection of vulnerable motor neurons [41]. Combining transcriptional screens on motor neurons which are differentially vulnerable in ALS and SMA can significantly refine the list of potential modifiers and lead to the identification of SNCA [42] and STMN1 [42, 43] as promising neuroprotective modifiers. These findings prove that comparing selectively resilient with vulnerable motor neurons can lead to the discovery of mechanisms that drive protection. It also demonstrates that combining findings from different disease models increases the likelihood of effective identification of promising disease modifiers. Uncovering the similarities and differences in selective vulnerability patterns across models can provide a new platform to uncover the mechanism driving motor neuron resistance and susceptibility.

In this study, we performed a thorough body-wide analysis of selectively vulnerable patterns in the *Smn*^*2B/-*^ mouse model. We demonstrate that the *Smn*^*2B/-*^ mouse model displays a unique pattern of selective vulnerability, wherein the muscles from the core, proximal forelimbs and proximal hindlimbs display increased NMJ loss. We further show that this peripheral pathology correlates with an increased loss of motor neurons in the associated spinal cord segments. This regional pattern of vulnerability, where the proximal muscles are more vulnerable than the distal muscles, shows similarities to the patterns of vulnerability observed in patients [21–23]. We subsequently assembled previously published results from selectively vulnerable muscle analyses from the *Smn*^*-/-*^*;SMN2,* Taiwanese and *SMNΔ7* mouse models and used a seven-point colour-coded classification tool to compare patterns of selective vulnerability within and between models. While most models (with the notable exception of the Taiwanese mouse model) displayed denervation in a range of muscles, they each showed unique patterns of selective vulnerability. For example, NMJs of facial motor neurons were highly vulnerable in the *SMNΔ7* mouse model, while completely protected in the *Smn*^*2B/-*^ mouse model. To further identify groups of transcripts that have the potential to drive motor neuron protection, we performed cross-model comparative bioinformatics analysis and enrichment analysis utilising published RNA-seq data on facial motor neurons from *Smn*^*2B/-*^ and *SMNΔ7* mice. We demonstrate that an increase in transcripts associated with protein localisation to cell periphery and ribonucleoprotein complex biogenesis correlated with protection from degeneration. We propose that these transcript clusters could contribute to the protection of motor neurons in SMA mouse models.

## Materials and methods

### Mouse maintenance

*Smn* ^*2B/2B*^ mice [22; C57Bl/6J congenic backgound] were interbred with *Smn*^*+/−*^ (Jackson Laboratories strain formerly 010921, now 006214, C57Bl/6 congenic background) to obtain *Smn*^*2B/+*^ control mice and *Smn*^*2B/-*^ experimental mice. *Smn*^*+/-*^*;SMN2*^*2copy*^*;SMNΔ7*^*2copy*^ (Jackson strain 005025, FVB congenic background) mice were interbred to produce *Smn*^*-/-*^*;SMN2*^*2copy*^*;SMNΔ7*^*2copy*^ (aka *SMN∆7*) mice. All mice were maintained on a 12-h light/dark cycle under pathogen-free conditions within animal facilities of the University of Edinburgh. All procedures were performed in accordance with the UK Home Office.

### Neuromuscular junction labelling and quantification

The muscles were immediately dissected from recently sacrificed mice and were fixed in 4% paraformaldehyde (PFA; Electron Microscopy Science) in PBS for 15 min. All muscles were microdissected post-fixation and permeabilised in 2% Triton X-100 (Sigma; PTB) in PBS for 30 min at room temperature. They were blocked in 4% bovine serum albumin (BSA)/1% Triton X-100 in PBS for 30 min followed by overnight incubation in primary antibodies (Neurofilament [NF; 2H3]; 1:50 and synaptic vesicle protein 2 [SV2]; 1:100 from Developmental Studies Hybridoma Bank) in blocking solution at 4 °C. Tetramethylrhodamine-conjugated α-bungarotoxin (BTX; 1:250; Thermo Fisher) in PBS was applied for 60 min at room temperature followed by PBS washes. The muscles were incubated in secondary antibody (Alexa Fluor 488 Rabbit anti-Mouse; Jackson ImmunoResearch Laboratories; 1:250) in PBS for 3–4 h in the dark at room temperature and washed with PBS. The muscles were whole-mounted on glass slides (Thermo Fisher) using Mowiol® (Calbiochem) and cover-slipped.

**Fig. 1:**
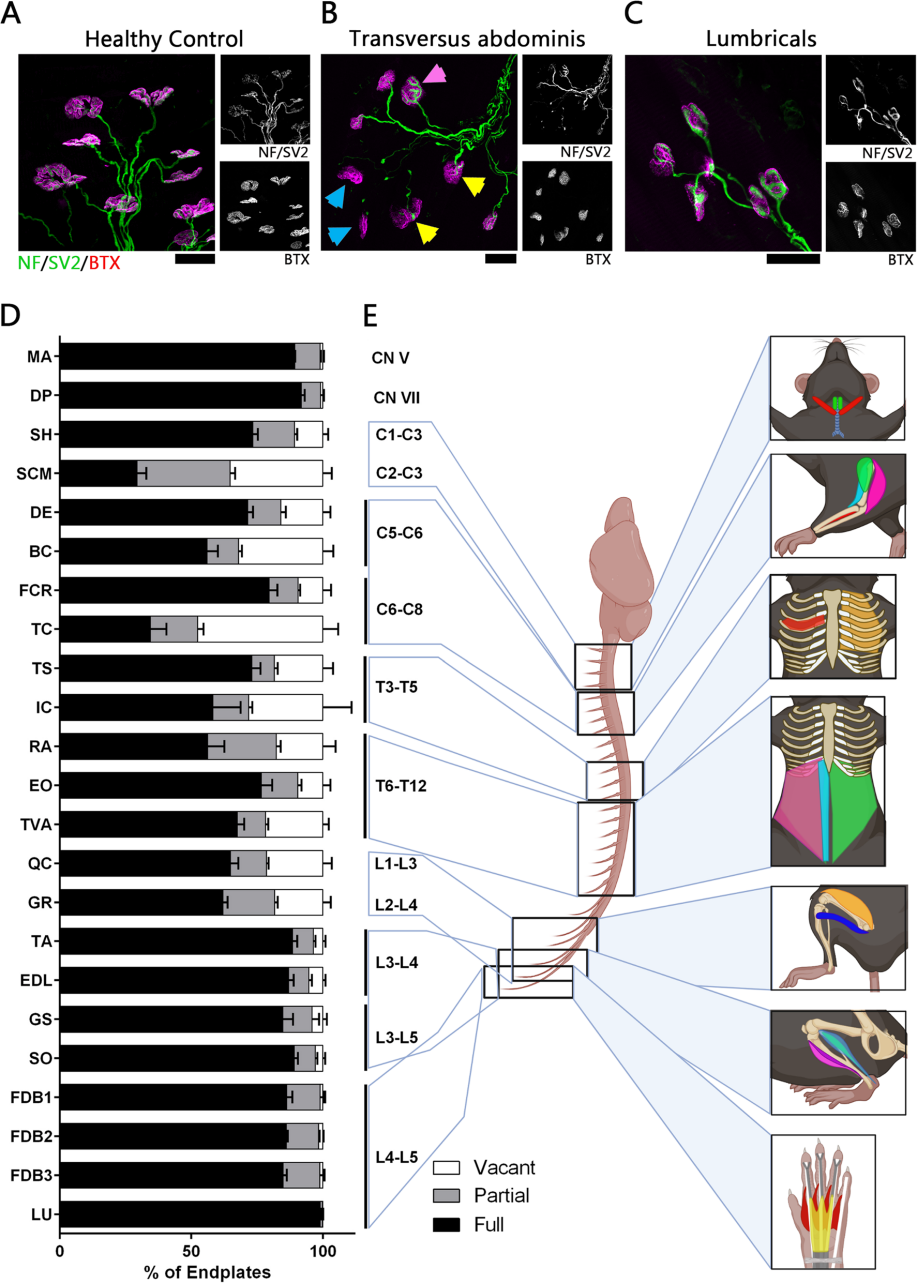
NMJ loss is more severe in muscles innervated by cervical, thoracic and upper lumbar regions of the spinal cord in *Smn*^*2B/−*^ mouse model at P16. **A**–**C** Representative confocal images showing NMJs labelled with antibodies against NF (green) and SV2 (green) and BTX (magenta) staining in a control muscle (*Smn*^*2B/+*^; gracilis), a vulnerable *Smn*^*2B/−*^ muscle (transversus abdominis) and a resistant *Smn*^*2B/−*^ muscle (lumbricals), respectively. Pink arrowheads indicate fully occupied endplates, yellow arrowheads show partially innervated endplates, and blue arrowheads indicate vacant endplates. Scale bar: 25μm. **D** Bar charts showing the quantification of percentage of full, partial and vacant endplates in *Smn*^*2B/−*^ mice in all investigated muscles. The muscles are displayed in order of their innervation from the cranial nerves and spinal cord segments, starting at the top with the muscles innervated by cranial nerves V (CN V) and VII (CN VII), followed by the muscles innervated from nerves arising from the cervical spinal cord and ending at the bottom with the muscles innervated by nerves arising from the lumbar spinal cord. The spinal cord segments that supply nerve innervation to the analysed muscles are indicated beside the respective bars. A high level of NMJ loss was visible across the muscles that are innervated by the nerves from cervical, thoracic and upper lumbar spinal cord segments. This pattern suggests that NMJ pathology starts at the centre of the spinal cord. *n*= 3 in IC and *n*=4 in all other muscles for *Smn*^*2B/−*^ mice. Error bars represent mean ± SEM. **E** Schematic diagram of the relationship between location of spinal cord segments and the anatomical location of the muscles innervated by the nerves arising from the indicated spinal cord segments, starting with the head muscles. Colours are for illustrative purposes only

Standard protocols for NMJ quantification were applied [14, 26–28, 42–45]. NMJ occupancy was quantified manually using a Leica-inverted fluorescent microscope at 40x magnification using filters that allow separate and simultaneous visualisation of green (NF, SV2) and red channels (BTX). All quantifications were undertaken blinded to the genotype of the muscle. A minimum of 100 endplates in three or more fields of view per muscle were quantified. The fields of view were chosen randomly across the muscle using the BTX staining. The analysed endplates were classified as vacant, partially occupied (where a pre-synaptic terminal only partially covers an AChR cluster) and fully occupied (see Fig. 1A for example). Representative example images are projections of z-stacks obtained by a Leica SP8 confocal microscope at ×63 magnification.

### Motor neuron labelling and quantification

The spinal cords were immediately dissected from recently sacrificed mice and fixed overnight in 4% paraformaldehyde (PFA; Electron Microscopy Science) in PBS. They were immersed in 30% sucrose in PBS for 48 h prior to embedding in 50% optimal cutting temperature compound (OCT)/15% sucrose in PBS. Spinal cord segments T5, T11 and L5 were transverse sectioned at 10 μM. Every tenth section was collected and labelled with Nissl/ChAT.

Sections were permeabilised in 0.3% Triton X-100 in PBS for 30 min and blocked in 0.3% Triton X-100/4% bovine serum albumin (BSA) in PBS for 60 min. Sections were incubated for three nights in ChAT (goat anti-choline acetyl-transferase; Merck Millipore; 1:100) primary antibody in a blocking solution at 4 °C. Slides were washed in 0.2% Triton X-100 in PBS and incubated in secondary antibody (AlexaFluor 555 Donkey anti-Goat; Life Technologies; 1:250) in a blocking solution for 2 h at room temperature. Sections were counterstained with DAPI (Life Technologies; 1:1000) and NeuroTrace® (Nissl; Life Technologies; 1:100) and mounted in Mowiol® (Calbiochem).

The total number of motor neuron cell bodies was quantified by fluorescent microscopy at 20x magnification using a Leica-inverted fluorescent microscope with filters that allow separate and simultaneous visualisation of green (Nissl) and red (ChAT) channels (example images shown in magenta green). All quantifications were undertaken blinded to the genotype of the sections. The number of ChAT-positive motor neurons in the ventral horn of the spinal cord was quantified bilaterally in every tenth section. Values that were missing due to sectioning artifacts were statistically imputed using the *mice: Multivariate Imputation by Chained Equations in R* [46] package in R studio. The values were assumed as missing at random (MAR) and were imputed using a predictive mean matching (PMM) imputation model. Five different values were predicted for each missing data point. The average from these five predicted values was used as the final imputed value for the following statistical analysis of the total number of motor neuron cell bodies in each spinal cord segment. Approximately, 12% of all values were imputed. Images were taken on a Leica DM8 fluorescent microscope at 20x magnification.

### Data acquisition and analysis for multi-model comparison of selective vulnerability patterns

Data on neuromuscular junction pathology in a range of muscles was obtained from previously published studies in four mouse models of SMA. Studies detailing NMJ pathology in *Smn*^*-/-*^*;SMN2*^*2copy*^ (herein referred to as *Smn*^*-/-*^*;SMN2*) [14, 24, 25], *Smn*^*Δ7/Δ7*^*;SMN2* aka Taiwanese [29], *SMNΔ7* [14, 20, 28] and *Smn*^*2B/-*^ [27] mouse models were utilised. Graphs showing the percentage of fully occupied endplates were exported from studies into Adobe Photoshop (version 21.2.0). A grid overlay was aligned to each graph’s axes to allow exact measurements of the mean percentage of fully occupied motor endplates.

Following the acquisition of the data, results were pooled by mouse model and the muscles were allocated to seven classification categories according to their percentage of fully occupied endplates at the end-stage of the disease. Each classification category was assigned a different colour (ranging from green, representing the muscles with minimal synaptic loss, to magenta representing the muscles with large amounts of NMJ loss) for representation in figures. The first six categories each include the muscles with percentages of fully occupied endplates within a 10% range, forming categories from 100 to 41% fully occupied endplates. The last category includes all the muscles that have 40% or less fully occupied endplates at the end-stage of the disease. All graphs were created using GraphPad Prism6.

### RNA-seq data processing and analysis

Three previously published RNA-seq data sets were re-analysed and cross-compared in this study. Raw data for *Smn*^*2B/-*^ mice and their controls were obtained via a kind agreement from the Kothary laboratory [27]), while raw data for *SMN∆7* mice and their controls was obtained from the Gene Expression Omnibus (Hedlund laboratory, GEO, accession number GSE115706; https://www.ncbi.nlm.nih.gov/geo/) and previously published in [41]. Our analysis compared differentially vulnerable motor neurons from cranial nucleus VII in the *Smn*^*2B/-*^ and *SMN∆7* mouse models of SMA. Moreover, we compared the CNVII derived from both models with *Smn*^*2B/-*^ thoracic spinal cord (tSC) from our previously published data set. Analysis was conducted on data from pre-symptomatic time-points in both models (P2 in *SMN∆7*; P10 in *Smn*^*2B/-*^).

The RNA-seq reads from both data sets were mapped to the 10-mm mouse genome assembly. Raw fastq data from the *Smn*^*2B/-*^ data sets were down-sampled to achieve a similar sequencing depth and mapping ratios across all samples of both data sets. Differential gene expression analysis was performed on both data set using DESeq2 package (v. 1.30.1) [47]. Only genes showing a log2 fold change difference of 1.5 between experimental and control genotype, and with an adjusted *p* value of *p*≤0.05 were considered for the analysis. Lists of differentially expressed genes (DEGs) were produced for both, *Smn*^*2B/-*^ and *SMN∆7* data sets and DEG overlaps between data sets were obtained. Overlapping DEGs were discarded, and all subsequent analyses were performed on the remaining DEGs of the *Smn*^*2B/-*^ CNVII data set only. Over-representation analysis on GO terms associated with the *Smn*^*2B/-*^ DEGs was conducted using clusterProfiler (v. 3.18.1). To visualise the expression profile of the GO terms associated genes between samples of both data sets, heatmaps of the *z* scores derived from normalized gene counts were generated. To retrieve networks of protein-protein interaction, we performed a STRING analysis (https://string-db.org/, v. 11.5 [48]) on all DEGs from the *Smn*^*2B/-*^ samples (using 433 genes as input), including interactions from database and experimental evidence, with at maximum of 5 interactions for each node of the first shell, and a medium confidence for interaction score to 0.04.

## Results

### Higher levels of synaptic loss are present at the centre of the spinal cord in the Smn^2B/−^ mouse model

Although some differentially vulnerable muscles have been identified in the *Smn*^*2B/-*^ mouse model of SMA [31], a thorough body wide analysis of the muscles was lacking. Therefore, we performed an extensive analysis of NMJ pathology of 20+ muscles located throughout the body in the *Smn*^*2B/−*^ mouse at the disease end-stage (P16). In our laboratory, this mouse displays a motor phenotype from around P10 and has a life expectancy of around 18 days.

NMJ pathology was analysed by quantification of the percentage of fully innervated, partially innervated, and vacant endplates in immuno-stained muscles from *Smn*^*2B/-*^ and control mice at P16 (Fig. 1A–C, See Supplementary Figure 1 for additional representative images). NMJ pathology was assessed in various muscles from different body regions. The analysis included two head muscles (digastric posterior [DP] and masseter [MA]), two neck muscles (sternocleidomastoid [SCM] and sternohyoid [SH]), two thoracic muscles (intercostals [IC] and triangularis sterni [TS]), three abdominal muscles (external oblique [EO], rectus abdominis [RA] and transversus abdominis [TVA]), four forelimb muscles (biceps brachii [BC], deltoid [DE], triceps brachii [TC] and flexor carpi radialis [FCR]), and eight hindlimb muscles (quadriceps [QC], gracilis [GR], extensor digitorum longus [EDL], gastrocnemius [GS], soleus [SO], tibialis anterior [TA], flexor digitorum brevis with its heads on digits 2, 3 and 4 [FDB2, FDB3 and FDB4] and hindlimb lumbricals [LU]). We ordered all investigated muscles by their level of innervation from the spinal cord (Fig. 1D, E). Analysis of the amount of synaptic loss showed that the cranial muscles had lower levels of NMJ loss with 91.8% fully innervated endplates remaining (digastric posterior [DP]). This is consistent with published work from the *Smn*^*2B/-*^ mouse on other cranial muscles showing no quantifiable denervation at disease end-stage [26, 27]. A higher amount of NMJ loss, with levels of fully innervated endplates as low as 29.3% (sternocleidomastoid [SCM]), could be observed in the neck muscles (SCM and SH) and forelimb muscles (BC, DE, TC and FCR) which are innervated by the cervical spinal nerves. There was also an increased vulnerability within the muscles that are innervated by the thoracic spinal nerves, namely in the thoracic muscles (IC and TS) and the abdominal muscles (EO, RA and TVA). A similar amount of vulnerability with 62% remaining fully innervated endplates (quadriceps [QC]) was also seen in the proximal leg muscles (GR and QC), which are innervated by the upper lumbar spinal nerves. Contrary to the proximal hindlimb muscles, the distal hindlimb muscles (TA, EDL, GS and SO) and foot muscles (FDB2,3,4 and LU), innervated by the middle and lower lumbar spinal nerves, did not show a large amount of synaptic loss. Between 84.8% (gastrocnemius [GS]) and 98.9% (lumbricals [LU]) innervation remained intact in these regions. These observations demonstrate a pattern of vulnerability consistent with increased susceptibility in the muscles of the core, neck and proximal limbs with lower levels of synaptic loss in the muscles of the distal limbs and face (Fig. 1D, E).

### Regional NMJ pathology correlates with motor neuron cell body loss in the spinal cord

**Fig. 2:**
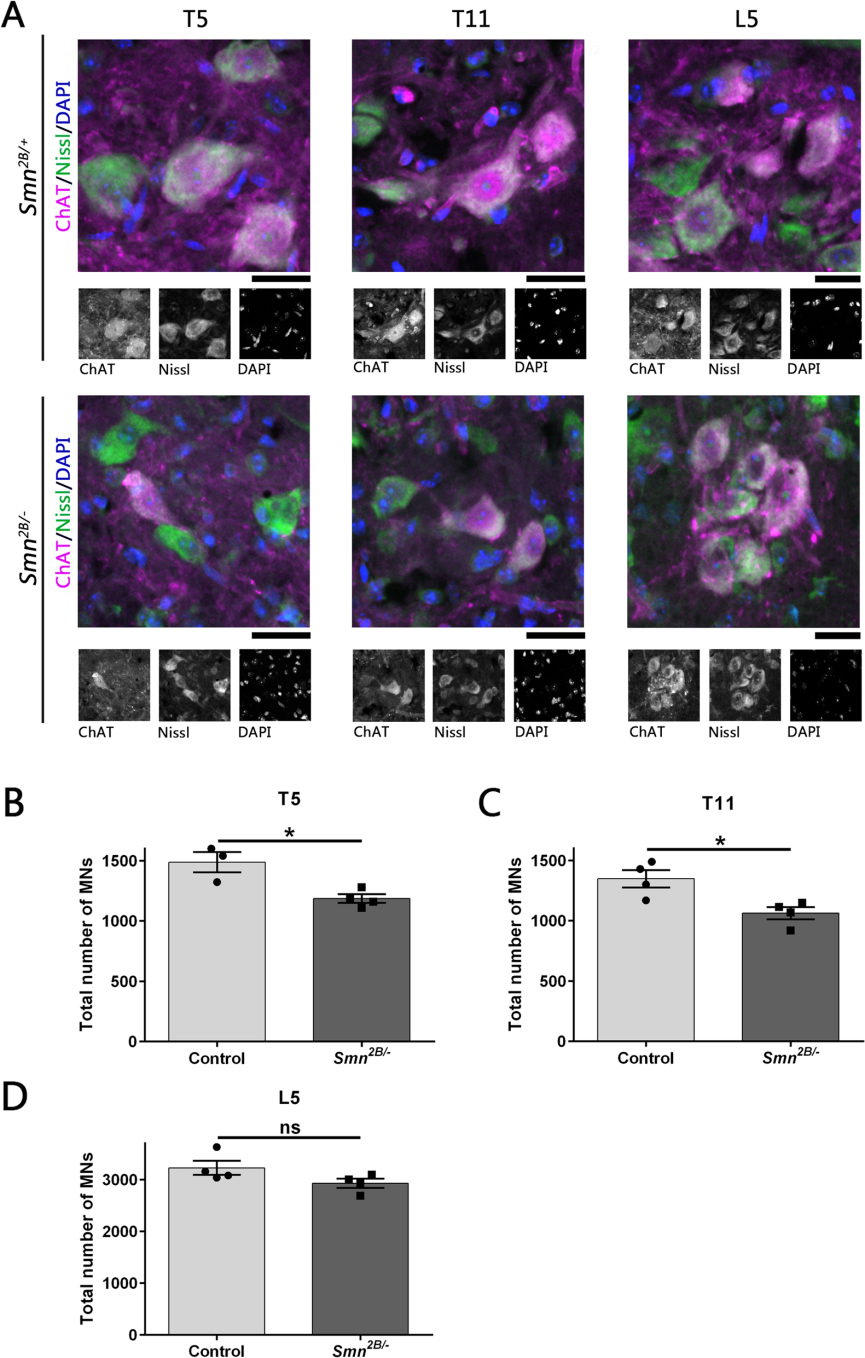
Significant motor neurons loss is observed in thoracic spinal cord segments in the *Smn*^*2B/−*^ mouse model. **A** Representative images showing motor neurons (MNs) labelled with an antibody against choline acetyltransferase (ChAT; magenta), as well as Nissl (green) and DAPI (blue) staining in *Smn*^*2B/−*^ and *Smn*^*2B/+*^ control mice in thoracic spinal cord segments (T5 and T11) and lumbar spinal cord segment L5 at P16. Scale bar: 50μm. **B**–**D** Bar charts showing the total number of MNs quantified in the ventral horn in the spinal cord segments T5, T11 and L5 in *Smn*^*2B/−*^ mice compared to *Smn*^*2B/+*^ controls. There was a significant decrease in the total number of MNs in *Smn*^*2B/−*^ mice compared to control in both thoracic spinal cord segments. However, there was no significant difference in the total number of MNs in the lumbar segment. Unpaired Student’s *t* test (ns= no significance, *p≤0.05). *n*=3,4 for controls and *Smn*^*2B/−*^ mice respectively in T5; *n*=4 for both genotypes in all other spinal cord segments. Error bars represent mean ± SEM

Data thus far suggests that the muscles innervated by the spinal nerves from the thoracic spinal cord are more vulnerable than the muscles innervated by the lower lumbar spinal nerves. We next investigated whether increased NMJ vulnerability correlated with corresponding motor neuron loss. We have previously shown that motor neuron loss can be detected in the *Smn*^*2B/−*^ mouse model from P15 and is preceded by NMJ loss [15]. We therefore quantified motor neuron somas throughout the ventral horn in T5, T11 and L5 spinal cord segments from P16 mice (Fig. 2). We observed a significant loss of motor neuron somas in T5 and T11 (*p*≤0.05). There was no significant difference in the total number of motor neurons at L5. These results indicate that NMJ loss is associated with the loss of motor neuron somas and provides further evidence that there is an increased vulnerability of motor neurons in the thoracic region compared to lower lumbar levels.

### Distinct patters of selective vulnerability in the Smn^-/-^;SMN2, Taiwanese, SMN∆7 and Smn^2B/−^ mouse models

Previous studies have utilised a range of available mouse models to classify the relative vulnerability of the muscles in SMA mouse models [14, 20, 24–29]. In order to gain insight into consistency of susceptibility patterns across SMA mouse models, we compared patterns of selectively vulnerable muscles across the four most commonly used SMA mouse models: the *Smn*^*-/-*^*;SMN2*, Taiwanese, *SMN∆7* and *Smn*^*2B/−*^ mouse model, supplemented by our own analysis from the *Smn*^*2B/-*^ mouse model (Fig. 1) and *SMN∆7* (Supplementary Figure 2) [14, 20, 24, 25, 27–29]. Towards this goal, we developed a seven-point colour-coded vulnerability classification tool. The muscles were classified based upon the percentage of fully innervated motor endplates, and each category was given a specific colour from dark green to dark magenta to represent a 10% range of fully occupied endplates (see legend in Fig. 4).

**Fig. 3:**
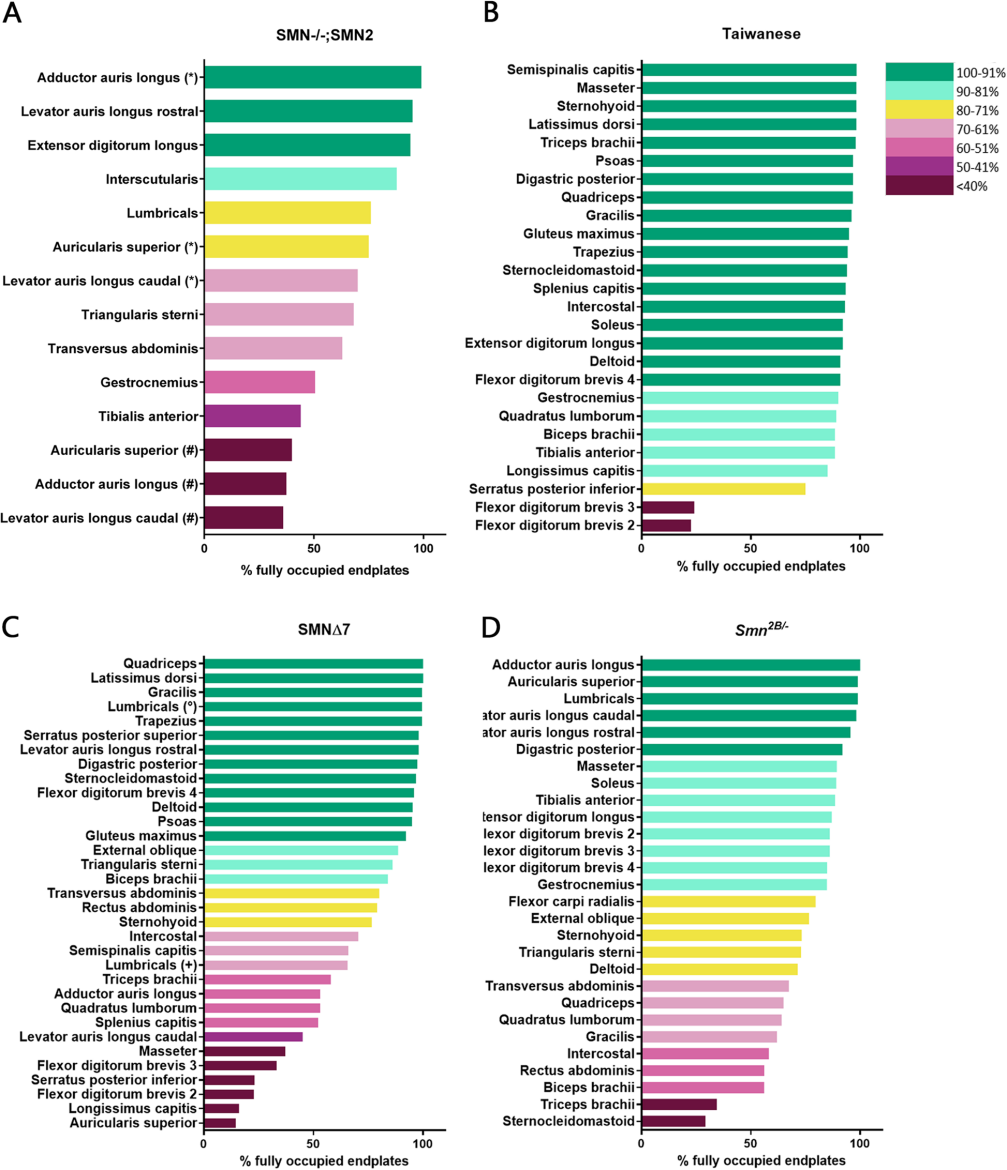
Comparison of NMJ loss in four mouse models indicates a similar range of vulnerability. **A**–**D** Bar charts showing the percentage of fully occupied endplates in previously investigated muscles in the *Smn*^*-/-*^*;SMN2* [14, 24, 25]; * = [14, 24], # = [25], Taiwanese [16, 29], *SMN∆7* [14, 16, 20, 28]; = [20]; + = [28] and *Smn*^*2B/-*^ [16, 27] mouse model, respectively. The muscles in each mouse model are ordered by decreasing percentage of fully occupied endplates. Each muscle is coloured according to its classification category by percentage of fully occupied motor endplates present at end-stage of disease. Each of the four mouse models displays a wide variability in the percentage of NMJ loss and therefore in vulnerability levels. Note that the percentage of fully occupied endplates ranges from almost 100% to lowest values of only around 20–30% in all four models

We found that in mouse models, the muscles appear to sit on a spectrum, displaying many different levels of vulnerability, rather than showing a bimodal split in relative vulnerability (Fig. 3). Furthermore, there appears to be a similar range in relative vulnerability across most models. In the *Smn*^*-/-*^*;SMN2* mouse model, vulnerability levels range from >99% of endplates remaining fully occupied in the adductor auris longus [14] to 36% remaining fully occupied endplates in the caudal band of the levator auris longus [25] (Fig. 3A). The percentage of innervated endplates in the *SMNΔ7* mouse models ranged from 100% in the quadriceps [20] to 15% in the auricularis superior (Fig. 3C, Supplementary Figure 2). In the *Smn*^*2B/−*^ mouse model, innervation ranged from 99% in the lumbrical muscles to 30% in the sternocleidomastoid (Fig. 3D). In the Taiwanese mouse model, the variability in vulnerability again followed a similar range (98% innervation in the semispinalis capitis to 23% innervation in the flexor digitorum brevis 2) [29]; Fig. 3B). However, it is notable that the majority of the muscles are comparatively resistant to synaptic loss in this model. These findings show that all four mouse models display a similar range of NMJ loss, but that the Taiwanese model shows a milder degree of NMJ pathology compared to the other three mouse models.

**Fig. 4:**
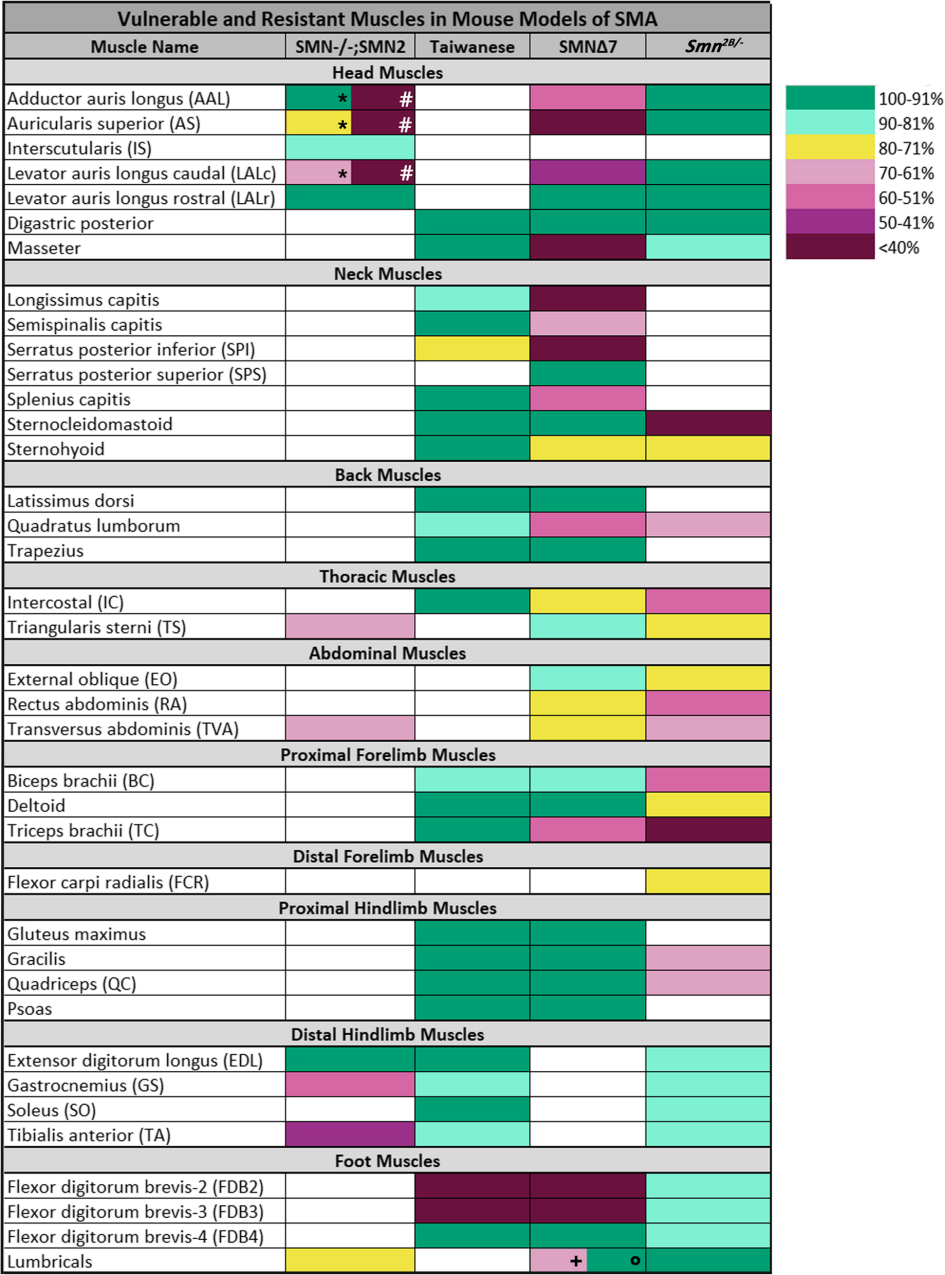
Comparison of NMJ loss in four mouse models indicates diverse patterns of selective vulnerability. The colour gradient legend describes seven classification categories of the NMJ pathology according to the percentage of fully occupied motor endplates in a muscle at disease end-stage (see ‘Methods’ section for more details). The table shows investigated muscles coloured according to their classification categories by percentage of fully occupied endplates in the *Smn*^*-/-*^*;SMN2* [14, 24, 25], Taiwanese [16, 29], *SMN∆7* [14, 16, 20, 28] and *Smn*^*2B/-*^ [16, 27] mouse model. The table displays the muscles categorised by body regions (head, neck, back, etc.). Note that for some muscles, different results were observed in two independent studies. This occurred in the AAL, AS and LALc muscles * = [14, 24]; # = [25] in the *Smn*^*-/-*^*;SMN2* model and the LU + = [28];° = [20] in the *SMN∆7* model. In the *Smn*^*-/-*^*;SMN2* mouse model, all body regions seem to be moderately affected and display different vulnerability levels, with the most severely affected the muscles in the hindlimb. The Taiwanese mouse model shows very little vulnerability, except for the FDB 2 and FDB 3. The vulnerability pattern in the *SMN∆7* mouse model shows that the head, neck and foot muscles are the most affected, followed by ventral core muscles (thoracoabdominal muscles) and the least vulnerability is seen in the limb and back muscles. The *Smn*^*2B/−*^ mouse model displays a regional vulnerability pattern, where the neck muscles, core muscles (thoracoabdominal muscles) and proximal fore- and hindlimbs seem more affected than the head muscles, distal limb and foot muscles

To gain a better understanding of the similarities and differences in vulnerability patterns of the mouse models, we used our analysis and available published data to compare specific muscles (Fig. 4). The table displays all previously investigated muscles in the *Smn*^*-/-*^*;SMN2*, Taiwanese, *SMN∆7* and *Smn*^*2B/−*^ mouse models, categorised by anatomical body regions. Each muscle in the table is displayed in the colour of the allocated category of our classification tool, based on its number of remaining fully occupied endplates (Fig. 4).

Analysis of this data by body region revealed that the vulnerability of a given muscle varied considerably between mouse models. Firstly, the regional pattern of vulnerability wherein there was an increase in synapse loss in core and proximal limb muscles which was evident in the *Smn*^*2B/−*^ mouse, was not seen in any of the other three models. Indeed, in the Taiwanese mouse model, the majority of muscles display between 100 and 81% fully innervated endplates. The only exceptions with severe NMJ loss were the FDB 2 and 3 of the foot muscles. In the *Smn*^*-/-*^*;SMN2* and *SMNΔ7* mouse models, many body regions include both vulnerable and resistant muscles. In the *SMN∆7* mouse model, the frequency of this dichotomy of vulnerability in certain body regions is particularly prominent and especially pronounced in the neck muscles. Three muscles are extremely vulnerable and two are moderately affected, whilst two are largely unaffected by pathology. In order to confirm that the variability in relative vulnerability was not due to observer effects, we quantified a range of muscles in the *SMNΔ7* and *Smn*^*2B/-*^ mice in our laboratory (Supplementary Figure 3). This verified the occurrence of differing levels of pathology between mouse models in the muscles such as the auricularis auris longus, auricularis superior, triangularis sterni, external oblique and rectus abdominis.

These profound differences between models can best be highlighted when looking at some examples of how individual muscles differ across models. For example, AS, AAL and LALc, three cranial muscles, are severely affected in the *SMNΔ7* model and the *Smn*^*-/-*^*;SMN2* mouse models, but resistant in the *Smn*^*2B/-*^ mouse model. In the Taiwanese model, almost all innervation is remaining in the TC muscle, while in the *SMN∆7* model, 57.9% endplates remain fully innervated, and only 34.4% endplates remain occupied in the *Smn*^*2B/−*^ mouse model. The TA muscle is >90% fully occupied in the *SMN∆7* and *Smn*^*2B/-*^ mouse models, but 44% fully occupied in the *Smn-/-;SMN2* mouse model. The IC muscles also behave differently across all models, where there is around 55% and 70% of NMJ remaining innervated in the *Smn*^*2B/−*^ model and *SMN∆7* mice, respectively, and almost no synaptic loss in the Taiwanese model.

In order to explore other factors which may affect relative vulnerability, the muscles were grouped based upon their fast synapsing (FaSyn) and delayed synapsing (DeSyn) phenotype, or muscle fibre type (Supplementary Figure 4). This revealed no correlation in any of the four models analysed.

These findings show that comparison of selective vulnerability patterns revealed four distinct patterns of selective vulnerability in commonly used mouse models of SMA. This discovery of profound differences in vulnerability patterns between mouse models is very important to consider during experimental design. It can also be a tool to better understand the mechanisms driving selective vulnerability in different mouse models of SMA. Further work is needed to uncover the mechanisms underlying NMJ and motor neuron vulnerability in these mouse models of disease.

### Transcriptional analysis on differentially vulnerable motor neurons reveals groups of transcripts that could contribute to motor neuron protection

To investigate the molecular differences which may underlie the contrasting patterns of selective vulnerability, we conducted a cross-model transcriptional analysis of differentially vulnerable motor neurons. We focused on facial motor neurons as their innervation remains largely intact in the *Smn*^*2B/−*^ model, whereas in the *SMNΔ7* mouse model, >40% of the innervation is lost (Fig. 4). We therefore reasoned that unique transcriptional changes occurring in the facial motor neurons of the *Smn*^*2B/-*^ mouse may confer protection.

**Fig. 5:**
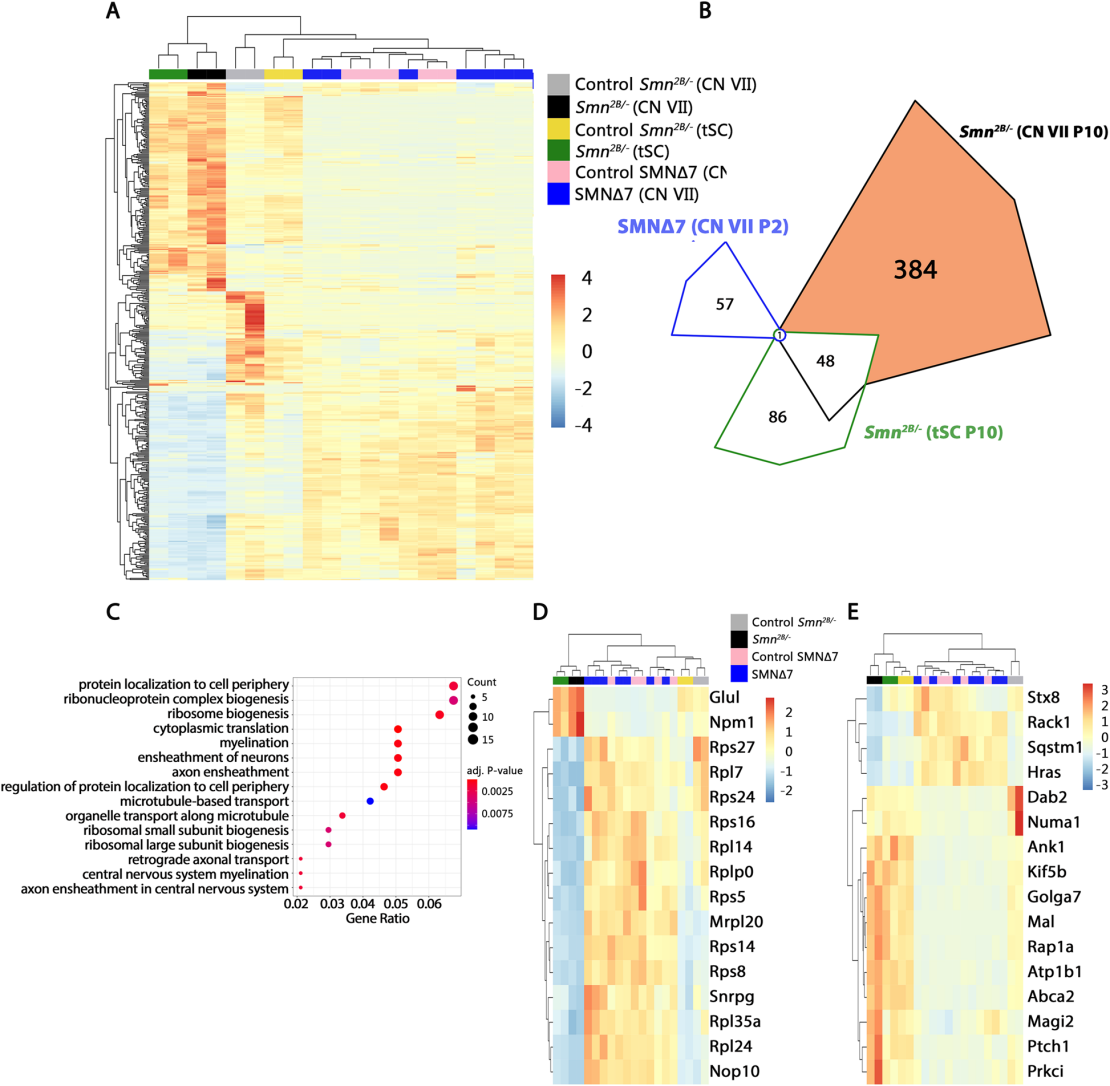
Transcriptional analysis shows enriched genes associated with rRNA processing, and vesicle trafficking and neuronal development. **A** Expression heatmap of the 433 DEGs between motor neurons from the CNVII in *Smn*^*2B/-*^. Relative expression levels in the thoracic spinal cord in *Smn*^*2B/-*^ and the CNVII in *SMN∆7* compared to their respective controls are also shown. Expression values were normalized and mean centred. **B** Venn diagram showing number of overlapping identified DEGs in all three motor neuron groups. Gene ontology analysis was done on changes found exclusively in the CNVII motor neurons from *Smn*^*2B/-*^ mice (384 DEGs). **C** Gene ontology (GO) term analysis for biological functions of the 384 differentially expressed genes in the CNVII of *Smn*^*2B/-*^ mice compared to CNVII of control mice. The size of the dot in the diagram reflects the number of genes included in the GO term. The colour of the dot shows the significance of the adjusted *p* value. Heatmaps with hierarchical clustering of **D** the 16 genes that are included in the GO terms ‘ribonucleoprotein complex biogenesis;’ **E** the 16 genes associated with the GO term cluster ‘protein localisation to cell periphery’. In both heatmaps, *Z* scores are calculated and plotted instead of the normalized expression value only. Note that in both heatmaps the *Smn*^*2B/-*^ *CNVII* samples are altered in comparison to their controls, while there is no difference between *SMN∆7* and their controls

In previous studies, we have individually performed transcriptional analysis on facial motor neurons from *Smn*^*2B/-*^ mice atP10 or *SMNΔ7* mice at P2, P5 and P10 compared to their respective controls [27, 41]. Here, we cross-compared the data sets at a comparable pre-symptomatic time-point (P10 in the *Smn*^*2B/-*^ and P2 in the *SMN∆7* mouse model) and compiled a list of DEGs in the resistant CNVII motor neurons in the *Smn*^*2B/-*^ model (Fig. 5A).

Hierarchical clustering using the DEGs of the resistant CNVII in the *Smn*^*2B/-*^ model showed a clear separation of the altered DEGs between this model and the *SMNΔ7* model (Fig. 5A).

We then compiled a group of 384 DEGs which were unique to the *Smn*^*2B/-*^ CNVII data set, by removing any transcripts which were also altered in *Smn*^*2B/-*^ vulnerable tSC motor neurons (48 transcripts) or *SMNΔ7* CNVII motor neurons (1 transcript) compared to their respective controls (Fig. 5B). To interpret the biological processes dysregulated in *Smn*^*2B/-*^ facial motor neurons, we used the 384 DEGs unique to these and conducted a Gene Ontology (GO) term enrichment analysis (Fig. 5A). We identified a number of GO terms, including ‘protein localisation to cell periphery’ [GO:1904375] and ‘ribonucleoprotein complex biogenesis’ [GO:0022613] (Fig. 5D, E). The ‘ribonucleoprotein complex biogenesis’ GO term cluster included 16 transcripts and had an adjusted *p* value of *p*=0.004. Here, genes were associated with cell signalling, rRNA processing and maturation, and ribosome constituents and biogenesis (Fig. 5D). The GO term cluster ‘protein localisation to cell periphery’ included 16 differentially expressed transcripts and had an adjusted *p* value of *p*= 0.002. Genes in this cluster were associated with vesicle trafficking, microtubule dynamics and neuronal development (Fig. 5E).

**Fig. 6:**
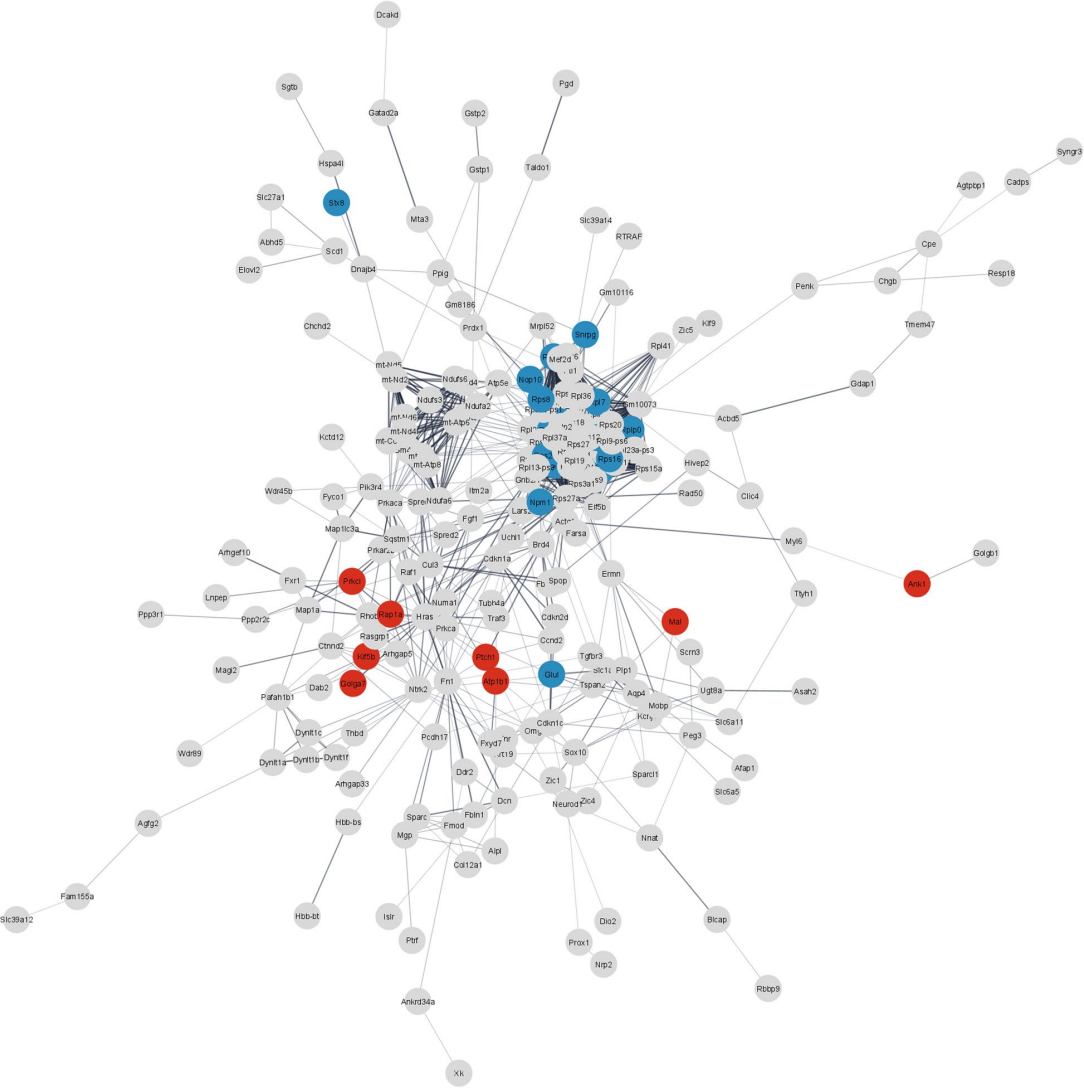
STRING analysis of DEGs from *Smn*^*2B/-*^ mouse model reveals large protein-protein interaction network. STRING analysis for protein-protein interactions including all differentially expressed genes regulated in the CNVII of the *Smn*^*2B/-*^ mouse model of SMA compared to control littermates. Nodes are coloured according to selected GO terms: (red) protein localisation to cell periphery and (blue) ribonucleoprotein complex biogenesis. Isolated nodes were removed. Network edges represent the confidence of the predicted associations between nodes (edge thickness = strength of data support)

In order to visualise the interaction between these networks and other dysregulated transcripts, we used the STRING database to retrieve networks of protein-protein interactions from all DEGs of *Smn*^*2B/-*^ CNVIII samples at P10 (Fig. 6). Ribosomal proteins from the small subunit (*Rps* proteins) and large subunit (*Rpl* proteins) formed a dense network, consistent with the GO term analysis. Most of the genes in this network contribute to ribosome biogenesis, including *Rps5, Rps16, Rpl24* and *Rps14*. Other genes like *Rps27, Rps24* and *Snrpg* contribute to pre-rRNA and rRNA processing and maturation. Among the DEGs with a role in vesicle trafficking, we found genes that are important for neuronal development and neurite outgrowth (*Magi2, Kif5b* and *Rap1a*), and microtubule dynamics (*Numa1, Kif5b* and *Prkci*). Furthermore, the network also included *Sqstm1*, which is connected to frontotemporal dementia and amyotrophic lateral sclerosis. Lastly, *Snrpg*, which was included in the GO term analysis, plays a role in RNA splicing as component of the SMN spliceosome assembly. In summary, we identified several transcriptional programs that are specifically activated in the facial motor neurons from *Smn*^*2B/-*^ animals and which may contribute to the protection of motor neurons in this model.

## Discussion

The findings in this study provide novel insights into the selective vulnerability patterns of mouse models of SMA and reveal groups of transcripts that could contribute to motor neuron protection. In this study, we have conducted a thorough body wide analysis of the selective vulnerability pattern present in the *Smn*^*2B/-*^ mouse model at the disease end-stage and show that the *Smn*^*2B/-*^ model displays a regional pattern of NMJ loss. Comparison of vulnerability patters in four SMA mouse models reveals dramatic differences in patterns of selective vulnerability between models. Finally, we show that an increase in transcripts associated with ribosomal bioenergetics, vesicle trafficking and neuronal development correlates with protection of motor neurons from degeneration.

### Contributing factors of differential vulnerability in the four most commonly used mouse models of SMA

Neuromuscular pathology within the *Smn*^*2B/-*^ mouse model displays a unique regional vulnerability pattern. This regional vulnerability pattern is a feature that was previously documented in SMA patients, where patients present with an increased vulnerability and earlier weakness in the core muscles, proximal leg and proximal arm muscles [21–23]. There is a common sparing of the muscles innervated by the facial nerve and distal lower limbs. We also observed an increased vulnerability of the proximal limbs compared to the distal limbs, and increased vulnerability of the core and neck muscles which has been observed in patients [21, 22]. A comprehensive study profiling patterns of vulnerability in patients will become increasingly pertinent, especially if we see the predicted correlation between relative vulnerability and treatment efficacy [34]. Elucidating patterns of selective vulnerability from autopsy material is not optimal, since the temporal order of motor neuron loss cannot be discerned. Due to clinical intervention and care, human autopsy material tends to reflect a later disease stage than mice at ‘end-stage’, which may be one reason why motor neuron loss in patients appears more profound than in mice. Regardless, based on the currently available data, the correlation between the regional patterns of vulnerability in SMA patients and the *Smn*^*2B/-*^ mouse model are intriguing and may support the use of this mouse model in the identification of protective disease modifiers of potential clinical relevance.

The analysis of synaptic loss in the *Smn*^*2B/-*^ mouse model presented here shows that the muscles innervated by cervical, thoracic and upper lumbar spinal nerves are more vulnerable than the muscles innervated by the cranial nerves and lower lumbar spinal nerves. The reasons for the selective vulnerability of this body region are unclear. Whilst motor unit type has been shown to be a key regulator of selective vulnerability in ALS [49, 50], there is no correlation between fibre type and relative vulnerability here, which is consistent with previous work in the *SMN∆7* mouse model [20]. Indeed, muscle fibre type does not correlate with relative vulnerability in any of the four mouse models examined. Further, it was suggested that the subdivision of NMJs with respect to their development and stability is a contributing factor to selective vulnerability [14, 51]. The muscles can be subdivided into fast synapsing (FaSyn) and delayed synapsing (DeSyn) muscles according to their intrinsically distinct features of the rate at which they acquire their organisation of mature NMJs [51]. The FaSyn muscles were found to be more vulnerable in the head muscles of an SMA mouse model than in the DeSyn muscles [14]. Our study rules out muscle fibre type or synapsing rate as key factors for motor neuron vulnerability. This is in line with previously published data from the *SMN∆7* mouse model, where some FaSyn muscles were also identified as resistant to pathology [20]. Lastly, it has been proposed that the location of motor neurons within the spinal cord could be responsible for determining their vulnerability in SMA, whereby those located medially, innervating axial and proximal limb muscles are more vulnerable that those located medially, innervating the distal limb [52, 53]. In support of this, in the *SMNΔ7* model, there was a higher degree of sensory motor breakdown at L1 (innervating proximal leg) than at L5 (distal leg) [52]. Analysis of motor neuron size in the *SMNΔ7* mouse also revealed a selective shrinkage of motor neurons innervating the proximal forelimb, compared to distal forelimb and phrenic motor neurons [53]. The current study shows that a similar pattern is present in the *Smn*^*2B/-*^ model, where vulnerability of muscles is most pronounced in the axial, proximal forelimb and proximal hindlimb muscles. Although this pattern does not bear out in the other mouse models of SMA, there appears to be an intriguing correlation between muscle location and relative vulnerability in at least the *Smn*^*2B/-*^ mouse model of SMA and in patients with SMA. It is often assumed that proximal motor units are larger than those found most distally. It is therefore tempting to speculate that increased motor unit size drives vulnerability. This idea has been previously discounted during a study on selective vulnerability in the *Smn*^*-/-*^*;SMN2* mouse model [25]. However, since the correlation between motor unit size and relative vulnerability was only performed on a small number of the muscles, all with comparatively small motor units, and did not include any axial or proximal limb muscles, this idea should be revisited.

### Dramatic difference in vulnerability patterns between mouse models of SMA

We compared the NMJ pathology of the four most commonly used mouse models of SMA and showed that each model displays a distinct pattern of selective vulnerability. This is consistent with a previous report where NMJ pathology in the tibialis anterior (TA) muscle and quadratus lumborum (QL) muscles were compared in three mouse models [16]. Whilst TA was consistently less vulnerable, levels of denervation in the QL varied between models from 0 to 55%. All of the investigated mouse models share a common mutation within the *Smn* gene to mimic the phenotype of human disease [31, 54–56]. Despite their shared mutation in the same gene, there is a drastic difference between the observed vulnerability patterns of the muscles between models and the reasons for this remain unclear. Comparing these different moue models of SMA and their divergent patterns of selective vulnerability therefore creates a valuable opportunity to further understand the mechanisms which drive motor neuron pathology in SMA. Whilst the mouse strains do vary between these models, it cannot explain the full extent of the disparities. Indeed, the Taiwanese model is on the same background as the *Smn*^*-/-*^*;SMN2* and *SMNΔ7* models, yet their pattern of NMJ loss is vastly different. This is consistent with previous reports where differences in mouse strain did not account for the variation in the severity of phenotype or degree of motor neuron loss observed between mouse models [16]. One possible explanation for this is relative Smn levels, which are higher in the Taiwanese compared to the *SMNΔ7* model. However, since Smn levels are similar between *Smn*^*2B/-*^ and Taiwanese mice, this cannot fully account for the disparities and further work will be required to discern why motor neuron pathology in less severe in this mouse model.

Previous work has identified important differences between mouse models of SMA. Motor neuron loss appears comparatively milder in the Taiwanese mouse model and this is consistent with our findings here (Figs. 3 and 4) [16, 25]. Other disparities between models have been previously noted, included selectively vulnerable regions of the spinal cord and P53 pathway activation [16]. This current work supports these important differences between models and highlight the need to consider the impact of the chosen model and experimental design on conclusions.

On possible explanation to account for the different patterns of vulnerability in the *Smn*^*2B/-*^ mouse compared to other models is postnatal age. Previous work has shown that postnatal age can influence the pathways responsible for axon degeneration and that different muscles mature at different rates [44, 45, 57]. During the first postnatal month, the neuromuscular system undergoes dynamic molecular and structural changes associated with postnatal maturation, including developmental synapse elimination and consolidation and maturation of remaining connections. During the time period, the rate of synaptic degeneration following traumatic or hypoxic injury accelerates [44, 45]. The acceleration in degeneration following injury has been attributed to postnatal changes in mitochondria, which are progressively enriched in both muscle and peripheral axons during the first postnatal month [57, 58]. Interestingly, bioenergetic status has also been proposed as a regulator of selective vulnerability in mouse models of SMA [40]. Developmental regulation of neuronal tissue dynamics could play an important role in the development of different vulnerability pattern. If we consider these multiple developmental process regulations, coupled with the fact that all models have a different age of onset, they pose a possible hypothesis as to why the patterns of selective vulnerability vary so drastically between models. Investigating how mitochondria change in the synapses, axons and cell bodies in the different motor units from different SMA mouse models could potentially shed light on the reasons for the differences in patterns of vulnerability between mouse models of SMA.

### Potentially important molecular pathways for motor neuron protection

By conducting a cross-model transcriptional analysis of motor neuron vulnerability, we show that certain groups of transcripts are candidate protective modifiers. Our analysis revealed an enrichment in transcripts associated with the GO term ‘ribonucleoprotein complex biogenesis’. Transcripts in this cluster contribute to ribosome biogenesis as part of the ribosomal proteins from the small and large subunit, as well as to pre-RNA and rRNA processing and maturation, and regulation of the translation apparatus. Since SMN and associated proteins have been implicated in both pre-mRNA splicing and in the regulation of translation [59–62], it is tempting to speculate that a change in the regulation of these pathways could account for why this subset of neurons are more resistant to a reduction in SMN levels. To further investigate this aspect, transcriptomics should be coupled with proteomics to delineate modifications in proteostasis that may counteract detrimental effects of SMN loss.

We also demonstrated an enrichment in the transcripts associated with the GO term ‘protein localisation to cell periphery’. Transcripts in this cluster contribute to cellular pathways concerned with vesicle trafficking, microtubule dynamics and neuronal development. *Rap1a, Prkci, Magi2, Hras*, and *Numa1* are all part of the *Ras* and *Rap1* signalling pathways. The Ras signalling pathway transduces signals from the extracellular milieu to the cell nucleus, activating specific genes for cell growth, division and differentiation. The *Rap1* signalling pathway controls cellular processes, such as cell migration, cell proliferation and cell survival. *Magi2, Kif5b, Sqstm1* and *Rap1a* are associated neuronal development and neurite outgrowth, and some have previously been implicated in the pathogenesis of neurodegenerative diseases. For example, *Kif5b* is an axonal kinesin involved in anterograde axonal transport. It is part of signalling pathways in various neurodegenerative diseases including Huntington’s disease, Parkinson’s disease and ALS. Another member of the kinesin family (*Kif5a*) contributes to cytoskeletal defects in the pathogenesis of ALS [63]. It has also been demonstrated that gene mutations associated with an ALS mouse model interfere with kinesin members and facilitate defects in axonal transport that ultimately lead to death of motor neurons [64]. We suggest that *Kif5b* and other kinesin members have the potential to also contribute to the pathogenesis of other motor neuron diseases like SMA. Furthermore, several of these transcripts, as mentioned, are important for axon function. Since SMN has pivotal functions in the axon and SMA is an axonopathy, several of these genes could be regulated as compensation for the loss of SMN to maintain axon function [65–69].

Another transcript in the enriched clusters of our analysis is *Sqstm1*. *Sqstm1* is further associated with mitophagy, the removal of damaged mitochondria through autophagy processes. The *Sqstm1* gene encodes p62, and mutations in the *Sqstm1* gene have previously been reported in both ALS and the related disorder frontotemporal dementia. The deficiency of p62 was associated with inhibited complex I mitochondrial respiration, due to deficits in the electron transport chain [70]. Silencing or knockout of p62 in animal models resulted in mitochondrial dysfunction [71–73]. These findings taken together demonstrate that dysfunction of the *Sqstm1* gene is strongly associated with pathophysiological events in energy metabolism. Since bioenergetics status was previously found to be implicated in motor neuron vulnerability [40], we suggest that upregulation of *Sqstm1* could be protective modifier of motor neuron pathology in SMA. Increasing the expression of Sqstm1 and other potential modifiers identified here in vivo will give further insight about their neuroprotective properties.

## Conclusions

In summary, the data presented above represents a detailed analysis of selective vulnerability pattern in four mouse models of SMA. Selective vulnerability patterns differ across all 4 mouse models, with the regional pattern in the *Smn*^*2B/-*^ mouse showing similarity to the regional pattern in SMA patients. Based on these findings, we identified populations of motor neurons that are differentially vulnerable in the *Smn*^*2B/-*^ and *SMN∆7* mouse model of SMA. We show that transcripts associated with cellular functions which are known to be perturbed in deficient motor neurons, such as RNA processing, axonal transport and bioenergetics status, are differentially regulated in selectively resistant neurons. These novel insights reveal promising targets that will be interesting to further investigate in the context of motor neuron protection in spinal muscular atrophy.

## Supplementary Information

**Additional file 1: Supplementary Figure 1.** Representative images from Smn2B/- mice. Confocal images from muscles showing NMJs labelled with antibodies against NF (green), SV2 (green) and BTX (red) from control (Smn2B/+) and Smn2B/- mice from muscles located in the head and neck (A,C), thoracoabdominal region (D,F), forelimb (G,I) and hind limb (J,N). Schematic images (B, E, H, K- M) show location of muscles, with colour linking to confocal images from specific muscles. Scale bar = 25um.

**Additional file 2: Supplementary Figure 2.** Analysis of synaptic loss in cranial and thoracoabdominal muscles in SMN∆7 mouse model at P12. (A) Schematic diagram of the anatomical locations of cranial muscles (adductor auris longus [AAL] and auricularis superior [AS]) in a dorsally viewed mouse. (B) Schematic diagram of the anatomical locations of thoracic muscles (triangularis sterni [TS]) in a mouse in supine position. (C) Schematic diagram of the anatomical locations of abdominal muscles (external oblique [EO] and rectus abdominis [RA]) in a mouse in supine position. (D-F) Bar charts showing the quantification of percentage of full, partial and vacant endplates in SMN∆7 mice compared to controls in cranial muscles (AAL and AS), thoracic muscles (TS), and abdominal muscles (EO and RA) respectively. Note that all cranial muscles show a significant increase in vacant motor endplates and a significant decrease in fully occupied endplates, whereas non of the thoracic or abdominal muscles show a significant increase in vacant endplates. Two-sided ANOVA with Sidak correction (ns= no significance, *p≤0.05, **p≤0.01, ***p≤0.001 and ****p≤0.0001). *n*=3 for controls and SMN∆7 mice respectively. Error bars represent mean ± SEM.

**Additional file 3: Supplementary Figure 3.** Comparison of levels of synaptic loss between the SMN∆7 mouse model and the Smn2B/− mouse model in muscles of three different body regions. (A-C) Bar charts showing the quantification of percentage of full, partial and vacant endplates in SMN∆7 mice compared to Smn2B/− mice in cranial muscles (adductor auris longus and auricularis superior), thoracic muscles (triangularis sterni), and abdominal muscles (external oblique and rectus abdominis) respectively. Note that all SMN∆7 cranial muscles show a significant increase in vacant motor endplates and a significant decrease in fully occupied endplates compared to the Smn2B/− cranial muscles, whereas Smn2B/− thoracic and abdominal muscles are significantly more vulnerable than in SMN∆7 mice. Significance levels indicate the statistical difference between full, partial and vacant endplates when comparing SMN∆7 to Smn2B/− mice. Two-sided ANOVA with Sidak correction (ns= no significance, *p≤0.05, **p≤0.01, ***p≤0.001 and ****p≤0.0001). *n*=3 for SMN∆7 mice, *n*=4-5 for Smn2B/− mice. Error bars represent mean ± SEM.

**Additional file 4: Supplementary Figure 4.** Comparison of intrinsic muscle properties in four mouse models of SMA. The colour gradient legend describes seven classification categories of neuromuscular junction pathology according to the percentage of fully occupied motor endplates in a muscle at disease end- stage (see methods section for more details). The tables shows investigated muscles coloured according to its classification categories by percentage of fully occupied endplates in the Smn -/- ;SMN2 (Murray et al., 2008; Murray et al., 2010; Thomson et al., 2012), Taiwanese (Lin et al., 2016), SMN∆7 (Murray et al., 2008; Ling et al., 2012; Comley et al., 2016) and Smn2B/- (Murray et al., 2015) mouse model. (A) The table displays the muscles categorised by delayed synapsing (DeSyn) or fast synapsing (FaSyn). Note that for the LALc muscle, different results were observed in two independent studies (* = (Murray et al., 2008); # = (Thomson et al., 2012)). (B) The table displays the muscles categorised by muscle fibre type (fast-twitch, mixed fibre types and slow-twitch). Note that for the AS, AAL, LALc and lumbrical muscles, different results were observed in two independent studies (* = (Murray et al., 2008); # = (Thomson et al., 2012); + = (Comley et al., 2016); ° = (Ling et al., 2012)).

## Data Availability

The *SMN∆7* mouse model RNA-seq dataset supporting the conclusions of this article is available in the Gene Expression Omnibus (GEO, accession number GSE115706). The *Smn*^*2B/-*^ mouse model RNA-seq datasets supporting the conclusions of this article is available from the corresponding author (LMM) on reasonable request.

## Abbreviations

ALS: Amyotrophic lateral sclerosis
AAL: Adductor auris longus
AS: Auricularis superior
BC: Biceps brachii
BTX: α-Bungarotoxin
CN VII: Cranial nerve nucleus VII
DE: Deltoid
DeSyn: Delayed synapsing
DEG: Differentially expressed gene
DP: Digastric posterior
EDL: Extensor digitorum longus
EO: External oblique
FaSyn: Fast synapsing
FCR: Flexor carpi radialis
FDB: Flexor digitorum brevis
GO: Gene Ontology
GR: Gracilis
GS: Gastrocnemius
IC: Intercostals
LALc: Levator auris longus caudal
LALr: Levator auris longus rostral
LU: Lumbricals
MA: Masseter
MN: Motor neuron
NF: Neurofilament
NMJ: Neuromuscular junction
QC: Quadriceps
RA: Rectus abdominis
SCM: Sternocleidomastoid
SH: Sternohyoid
SMA: Spinal muscular atrophy
SMN: Survival motor neuron protein
*SMN1*: Survival motor neuron 1 gene
SO: Soleus
TA: Tibialis anterior
TC: Triceps brachii
TS: Triangularis sterni
TVA: Transversus abdominis
