# Supplementary material for: Mouse models of SMA show divergent patterns of neuronal vulnerability and resilience
Source: Skelet Muscle. 2022 Sep 12;12:22. doi: 10.1186/s13395-022-00305-9 (PMC9465884; doi:10.1186/s13395-022-00305-9)
Supplement: Supplementary file 1 — Additional file 1: Supplementary Figure 1. Representative images from Smn2B/- mice. Confocal images from muscles showing NMJs labelled with antibodies against NF (green), SV2 (green) and BTX (red) from control (Smn2B/+) and Smn2B/- mice from muscles located in the head and neck (A,C), thoracoabdominal region (D,F), forelimb (G,I) and hind limb (J,N). Schematic images (B, E, H, K- M) show location of muscles, with colour linking to confocal images from specific muscles. Scale bar = 25um. [file 13395_2022_305_MOESM1_ESM.pdf]

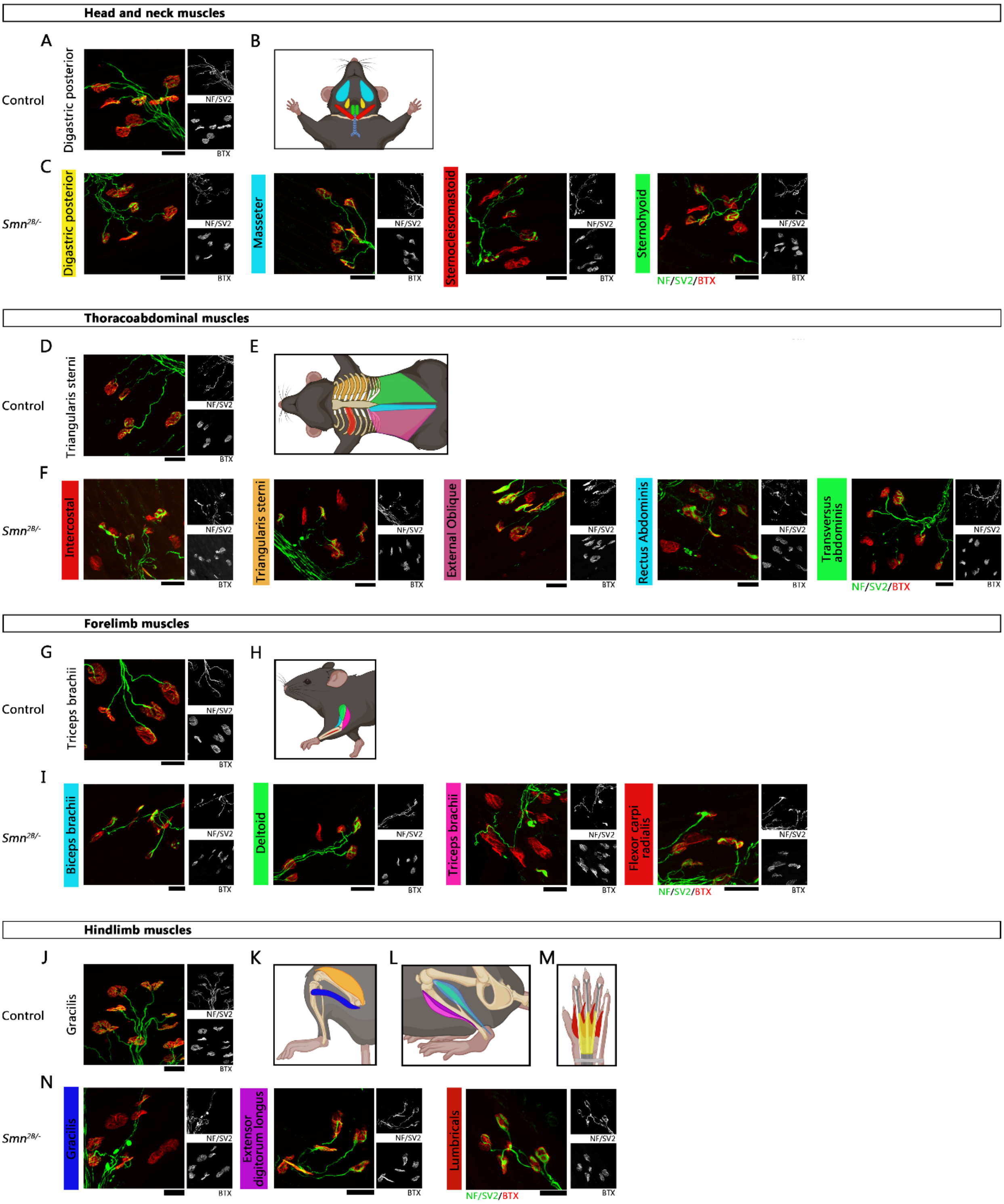

Supplementary Figure 1. Representative images from Smn2B/- mice. Confocal images from muscles showing NMJs labelled with antibodies against NF (green), SV2 (green) and BTX (red) from control (*Smn*$^{2B/+}$) and *Smn*$^{2B/-}$ mice from muscles located in the head and neck (A,C), thoracoabdominal region (D,F), forelimb (G,I) and hind limb (J,N). Schematic images (B, E, H, K- M) show location of muscles, with colour linking to confocal images from specific muscles. Scale bar = 25um.
