# Supplementary material for: Mouse models of SMA show divergent patterns of neuronal vulnerability and resilience
Source: Skelet Muscle. 2022 Sep 12;12:22. doi: 10.1186/s13395-022-00305-9 (PMC9465884; doi:10.1186/s13395-022-00305-9)
Supplement: Supplementary file 2 — Additional file 2: Supplementary Figure 2. Analysis of synaptic loss in cranial and thoracoabdominal muscles in SMN∆7 mouse model at P12. (A) Schematic diagram of the anatomical locations of cranial muscles (adductor auris longus [AAL] and auricularis superior [AS]) in a dorsally viewed mouse. (B) Schematic diagram of the anatomical locations of thoracic muscles (triangularis sterni [TS]) in a mouse in supine position. (C) Schematic diagram of the anatomical locations of abdominal muscles (external oblique [EO] and rectus abdominis [RA]) in a mouse in supine position. (D-F) Bar charts showing the quantification of percentage of full, partial and vacant endplates in SMN∆7 mice compared to controls in cranial muscles (AAL and AS), thoracic muscles (TS), and abdominal muscles (EO and RA) respectively. Note that all cranial muscles show a significant increase in vacant motor endplates and a significant decrease in fully occupied endplates, whereas non of the thoracic or abdominal muscles show a significant increase in vacant endplates. Two-sided ANOVA with Sidak correction (ns= no significance, *p≤0.05, **p≤0.01, ***p≤0.001 and ****p≤0.0001). n=3 for controls and SMN∆7 mice respectively. Error bars represent mean ± SEM. [file 13395_2022_305_MOESM2_ESM.pdf]

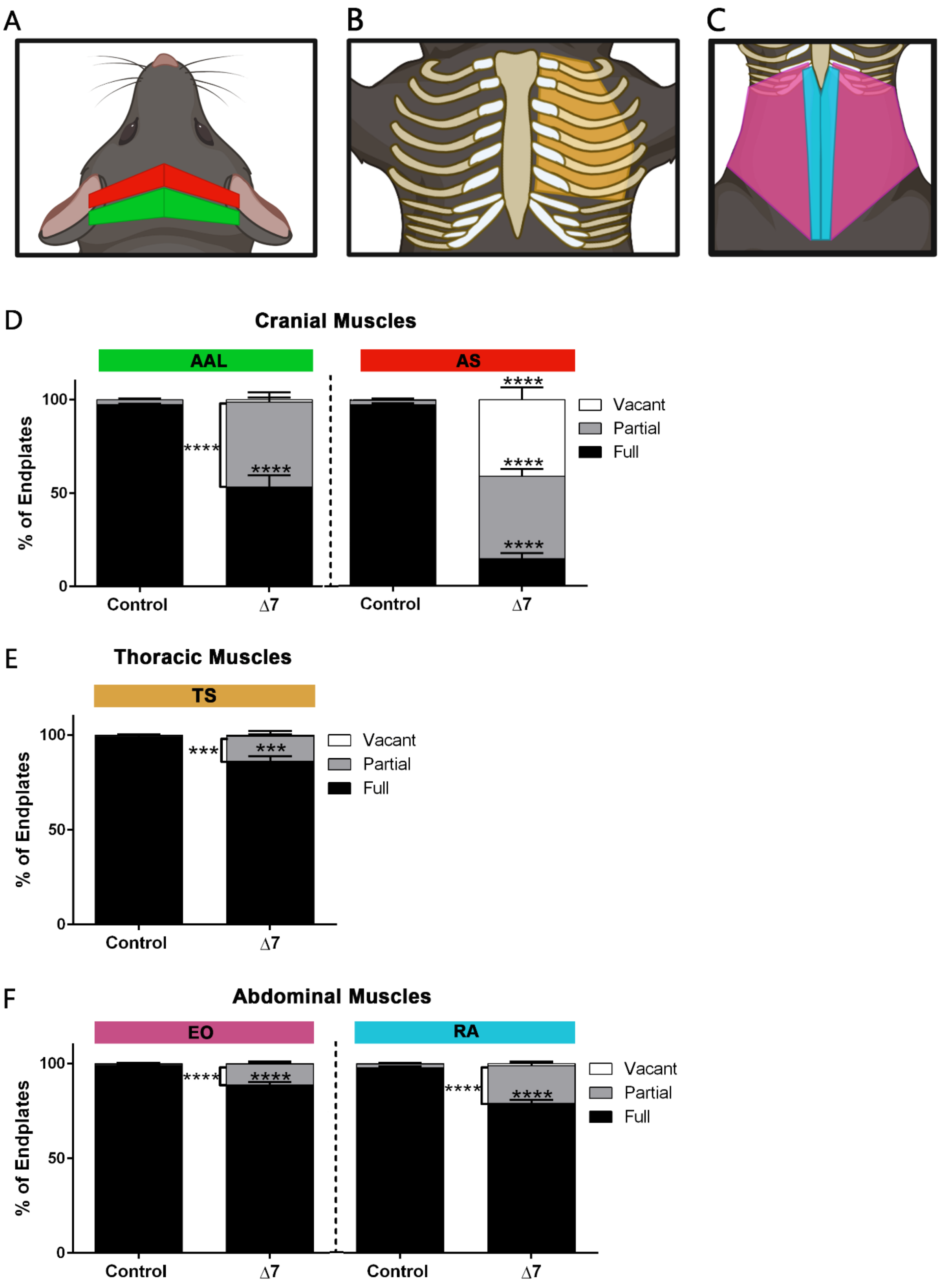

Supplementary Figure 2. Analysis of synaptic loss in cranial and thoracoabdominal muscles in SMNΔ7 mouse model at P12. (A) Schematic diagram of the anatomical locations of cranial muscles (adductor auris longus [AAL] and auricularis superior [AS]) in a dorsally viewed mouse. **(B)** Schematic diagram of

the anatomical locations of thoracic muscles (triangularis sterni [TS]) in a mouse in supine position. **(C)** Schematic diagram of the anatomical locations of abdominal muscles (external oblique [EO] and rectus abdominis [RA]) in a mouse in supine position. **(D-F)** Bar charts showing the quantification of percentage of full, partial and vacant endplates in SMNΔ7 mice compared to controls in cranial muscles (AAL and AS), thoracic muscles (TS), and abdominal muscles (EO and RA) respectively. Note that all cranial muscles show a significant increase in vacant motor endplates and a significant decrease in fully occupied endplates, whereas non of the thoracic or abdominal muscles show a significant increase in vacant endplates. Two-sided ANOVA with Sidak correction (ns= no significance, *p≤0.05, **p≤0.01, ***p≤0.001 and ****p≤0.0001). n=3 for controls and SMNΔ7 mice respectively. Error bars represent mean ± SEM.
