# Supplementary material for: Mouse models of SMA show divergent patterns of neuronal vulnerability and resilience
Source: Skelet Muscle. 2022 Sep 12;12:22. doi: 10.1186/s13395-022-00305-9 (PMC9465884; doi:10.1186/s13395-022-00305-9)
Supplement: Supplementary file 3 — Additional file 3: Supplementary Figure 3. Comparison of levels of synaptic loss between the SMN∆7 mouse model and the Smn2B/− mouse model in muscles of three different body regions. (A-C) Bar charts showing the quantification of percentage of full, partial and vacant endplates in SMN∆7 mice compared to Smn2B/− mice in cranial muscles (adductor auris longus and auricularis superior), thoracic muscles (triangularis sterni), and abdominal muscles (external oblique and rectus abdominis) respectively. Note that all SMN∆7 cranial muscles show a significant increase in vacant motor endplates and a significant decrease in fully occupied endplates compared to the Smn2B/− cranial muscles, whereas Smn2B/− thoracic and abdominal muscles are significantly more vulnerable than in SMN∆7 mice. Significance levels indicate the statistical difference between full, partial and vacant endplates when comparing SMN∆7 to Smn2B/− mice. Two-sided ANOVA with Sidak correction (ns= no significance, *p≤0.05, **p≤0.01, ***p≤0.001 and ****p≤0.0001). n=3 for SMN∆7 mice, n=4-5 for Smn2B/− mice. Error bars represent mean ± SEM. [file 13395_2022_305_MOESM3_ESM.pdf]

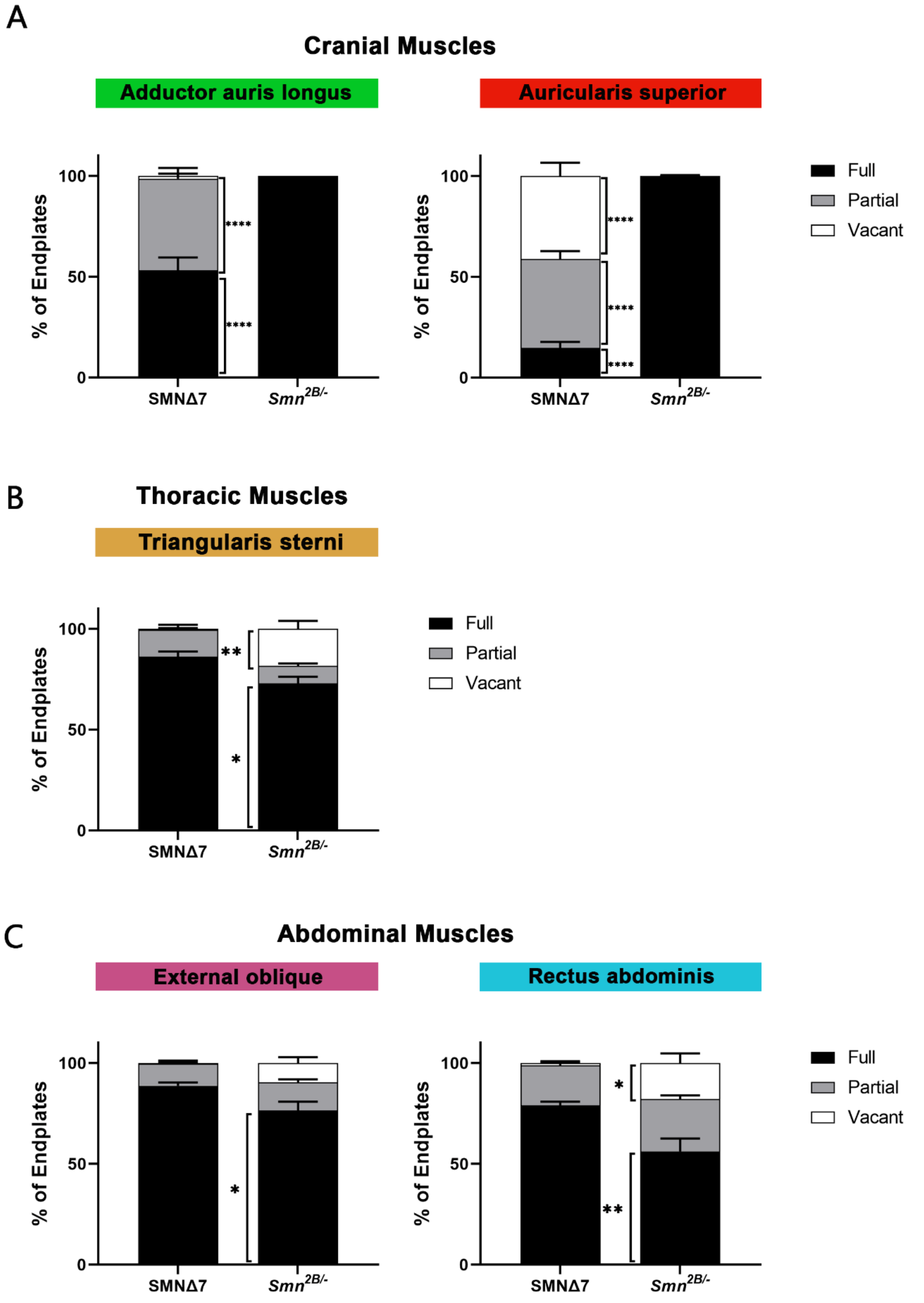

Supplementary Figure 3. Comparison of levels of synaptic loss between the SMNΔ7 mouse model and the *Smn*$^{2B/-}$ mouse model in muscles of three different body regions. (A-C) Bar charts showing

the quantification of percentage of full, partial and vacant endplates in SMNΔ7 mice compared to *Smn*$^{2B/-}$ mice in cranial muscles (adductor auris longus and auricularis superior), thoracic muscles (triangularis sterni), and abdominal muscles (external oblique and rectus abdominis) respectively. Note that all SMNΔ7 cranial muscles show a significant increase in vacant motor endplates and a significant decrease in fully occupied endplates compared to the *Smn*$^{2B/-}$ cranial muscles, whereas *Smn*$^{2B/-}$ thoracic and abdominal muscles are significantly more vulnerable than in SMNΔ7 mice. Significance levels indicate the statistical difference between full, partial and vacant endplates when comparing SMNΔ7 to *Smn*$^{2B/-}$ mice. Two-sided ANOVA with Sidak correction (ns= no significance, *$p \leq 0.05$, **$p \leq 0.01$, ***$p \leq 0.001$ and ****$p \leq 0.0001$). n=3 for SMNΔ7 mice, n=4-5 for *Smn*$^{2B/-}$ mice. Error bars represent mean ± SEM.
