# Supplementary material for: Mouse models of SMA show divergent patterns of neuronal vulnerability and resilience
Source: Skelet Muscle. 2022 Sep 12;12:22. doi: 10.1186/s13395-022-00305-9 (PMC9465884; doi:10.1186/s13395-022-00305-9)
Supplement: Supplementary file 4 — Additional file 4: Supplementary Figure 4. Comparison of intrinsic muscle properties in four mouse models of SMA. The colour gradient legend describes seven classification categories of neuromuscular junction pathology according to the percentage of fully occupied motor endplates in a muscle at disease end- stage (see methods section for more details). The tables shows investigated muscles coloured according to its classification categories by percentage of fully occupied endplates in the Smn -/- ;SMN2 (Murray et al., 2008; Murray et al., 2010; Thomson et al., 2012), Taiwanese (Lin et al., 2016), SMN∆7 (Murray et al., 2008; Ling et al., 2012; Comley et al., 2016) and Smn2B/- (Murray et al., 2015) mouse model. (A) The table displays the muscles categorised by delayed synapsing (DeSyn) or fast synapsing (FaSyn). Note that for the LALc muscle, different results were observed in two independent studies (* = (Murray et al., 2008); # = (Thomson et al., 2012)). (B) The table displays the muscles categorised by muscle fibre type (fast-twitch, mixed fibre types and slow-twitch). Note that for the AS, AAL, LALc and lumbrical muscles, different results were observed in two independent studies (* = (Murray et al., 2008); # = (Thomson et al., 2012); + = (Comley et al., 2016); ° = (Ling et al., 2012)). [file 13395_2022_305_MOESM4_ESM.pdf]

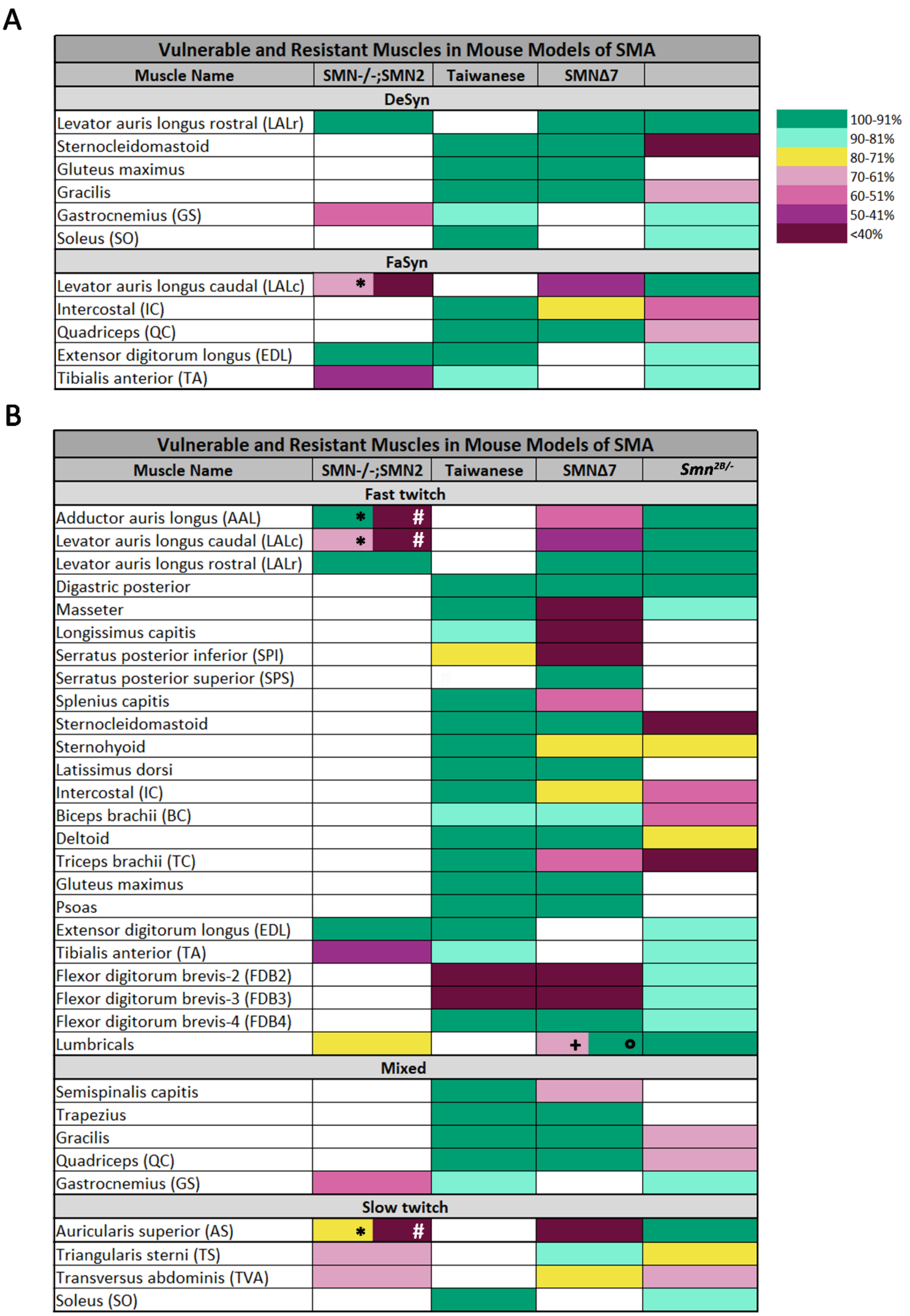

Supplementary Figure 4. Comparison of intrinsic muscle properties in four mouse models of SMA.

The colour gradient legend describes seven classification categories of neuromuscular junction pathology according to the percentage of fully occupied motor endplates in a muscle at disease end-

stage (see methods section for more details). The tables shows investigated muscles coloured according to its classification categories by percentage of fully occupied endplates in the *Smn*$^{-/-}$*;SMN2* (Murray et al., 2008; Murray et al., 2010; Thomson et al., 2012), Taiwanese (Lin et al., 2016), SMNΔ7 (Murray et al., 2008; Ling et al., 2012; Comley et al., 2016) and *Smn*$^{2B/-}$ (Murray et al., 2015) mouse model. **(A)** The table displays the muscles categorised by delayed synapsing (DeSyn) or fast synapsing (FaSyn). Note that for the LALc muscle, different results were observed in two independent studies (* = (Murray et al., 2008); # = (Thomson et al., 2012)). **(B)** The table displays the muscles categorised by muscle fibre type (fast-twitch, mixed fibre types and slow-twitch). Note that for the AS, AAL, LALc and lumbrical muscles, different results were observed in two independent studies (* = (Murray et al., 2008); # = (Thomson et al., 2012); + = (Comley et al., 2016); ° = (Ling et al., 2012)).
